# Using Social Media to Mine and Analyze Public Opinion Related to COVID-19 in China

**DOI:** 10.3390/ijerph17082788

**Published:** 2020-04-17

**Authors:** Xuehua Han, Juanle Wang, Min Zhang, Xiaojie Wang

**Affiliations:** 1 State Key Laboratory of Resources and Environmental Information System, Institute of Geographic Sciences and Natural Resources Research, Chinese Academy of Sciences, Beijing 100101, China; hanxh@lreis.ac.cn (X.H.); zhangmin@lreis.ac.cn (M.Z.); wxj@lreis.ac.cn (X.W.); 2 College of Resources and Environment, University of Chinese Academy of Sciences, Beijing 100049, China; 3 Jiangsu Center for Collaborative Innovation in Geographical Information Resource Development and Application, Nanjing 210023, China; 4 School of Civil and Architectural Engineering, Shandong University of Technology, Zibo 255049, China

**Keywords:** coronavirus, COVID-19, social media, public opinion, China, resource allocation

## Abstract

The outbreak of Corona Virus Disease 2019 (COVID-19) is a grave global public health emergency. Nowadays, social media has become the main channel through which the public can obtain information and express their opinions and feelings. This study explored public opinion in the early stages of COVID-19 in China by analyzing Sina-Weibo (a Twitter-like microblogging system in China) texts in terms of space, time, and content. Temporal changes within one-hour intervals and the spatial distribution of COVID-19-related Weibo texts were analyzed. Based on the latent Dirichlet allocation model and the random forest algorithm, a topic extraction and classification model was developed to hierarchically identify seven COVID-19-relevant topics and 13 sub-topics from Weibo texts. The results indicate that the number of Weibo texts varied over time for different topics and sub-topics corresponding with the different developmental stages of the event. The spatial distribution of COVID-19-relevant Weibo was mainly concentrated in Wuhan, Beijing-Tianjin-Hebei, the Yangtze River Delta, the Pearl River Delta, and the Chengdu-Chongqing urban agglomeration. There is a synchronization between frequent daily discussions on Weibo and the trend of the COVID-19 outbreak in the real world. Public response is very sensitive to the epidemic and significant social events, especially in urban agglomerations with convenient transportation and a large population. The timely dissemination and updating of epidemic-related information and the popularization of such information by the government can contribute to stabilizing public sentiments. However, the surge of public demand and the hysteresis of social support demonstrated that the allocation of medical resources was under enormous pressure in the early stage of the epidemic. It is suggested that the government should strengthen the response in terms of public opinion and epidemic prevention and exert control in key epidemic areas, urban agglomerations, and transboundary areas at the province level. In controlling the crisis, accurate response countermeasures should be formulated following public help demands. The findings can help government and emergency agencies to better understand the public opinion and sentiments towards COVID-19, to accelerate emergency responses, and to support post-disaster management.

## 1. Introduction

As of March 7 2020, the global number of confirmed cases of Corona Virus Disease 2019 (COVID-19) surpassed 100,000, covering more than 100 countries [1]. COVID-19 is a respiratory disease caused by a novel coronavirus. Since 8 December 019, clusters of pneumonia cases of unknown etiology emerged in Wuhan City, Hubei Province, China [2]. Mass transmission occurred in a matter of weeks in Wuhan, and people in the resulting chains of transmission spread the infection by traveling during the Chinese New Year holidays in early 2020. As a growing number of confirmed cases of infections was reported, the Chinese government took prompt response measures to combat the virus. On 9 January 2020, the causative agent of this pneumonia was initially confirmed as a novel coronavirus. On 20 January the National Health Commission of the People’s Republic of China (NHFPC) classified COVID-19 as a category B infectious disease based on the Law on Prevention and Control of Infectious Diseases and took preventive and control measures for category A infectious diseases [3]. On 23 January Wuhan City was put under lockdown to contain the outbreak [4]. On February 2, Huoshenshan Hospital in Wuhan, a 1000-bed makeshift hospital for treating infected patients, was built just in 10 days [5]. On 8 February China completed work on Leishenshan Hospital, another 1600-bed makeshift hospital in Wuhan [6]. As of 10 February the number of confirmed cases of COVID-19 in China surpassed 42,000 [7]. In the early stages of the COVID-19 outbreak in China, there was a fierce race between the growth of patients and the allocation of medical resources.

Coinciding with the Lunar New Year Festival, COVID-19 not only disrupted peoples’ normal lives, it also attracted great attention from all circles of society. In these circumstances, billions of people eagerly acquired information about COVID-19 through social media. Social media not only provides a platform for sharing personal lives but can also be used to examine public opinion and perceptions. This source of information may be comparable to the public comments collected by traditional approaches (e.g., questionnaires) [8]. Topics and sentiments related to COVID-19 spread rapidly, thus influencing public behavior during the epidemic. Combined with spatiotemporal information collected from social media, analyses of public opinion on COVID-19 are important for improving emergency responses, enhancing sentiment awareness, and supporting decision making [9].

However, public opinion on social media has the characteristics of irrationality, strong infectivity, and conformity. With the proliferation of web-based social and communication technologies, the wide use of social media has enabled broad public participation in emergency response [10,11]. A growing number of people use location-based social network services (e.g., Twitter, Facebook, or Sina-Weibo), with which they create time-stamped, geo-located data and share information about their immediate surroundings [12]. The rapid development of machine learning and text mining facilitates the analysis and understanding of human behavior, public responses, potential courses of action, and public opinions from social media data in emergency situations [13,14]. Gruebner et al. (2018) extracted the negative emotions of people in New York City before, during, and after superstorm Sandy from Twitter data using an advanced sentiment analysis method called “Extracting the Meaning Of Terse Information in a Visualization of Emotion” (EMOTIVE) [15]. Dahal used the Latent Dirichlet Allocation (LDA) topic model and sentiment analysis model to extract topics and calculate the sentiment index from Twitter data related to climate change [16]. Wang analyzed the temporal-spatial characteristics, topic distribution, and retweet network of Twitter data related to wildfires via kernel density estimation (KDE), text mining, and social network analysis [17]. Ye divided Weibo texts related to dengue fever into five topics and found that the temporal-spatial characteristics of Weibo texts were related to the dynamics of dengue fever based on LDA [18]. Zong conducted content analysis and temporal analysis on tweets related to chemical plant explosions in Tianjin, China [19]. These progresses in social media mining and analysis enabled the researchers to obtain public opinions in the early stages of COVID-19.

This study aimed to identify public opinion during the COVID-19 outbreak from social media and analyze its spatial-temporal characteristics from 9 January to 10 February 2020 in China. A topic extraction and classification model was built to identify the topics of COVID-19-related Weibo texts and uncover public sentiments in response to COVID-19. A series of topics and temporal and spatial distributions were identified and discussed.

## 2. Data and Methods

### 2.1. Data and Data Pre-Processing

Sina-Weibo (http://us.Weibo.com), often referred to as Weibo, is one of the most popular social media platforms in China. Weibo had over 516 million active users each month in 2019. This study acquired Weibo texts related to COVID-19. Using Weibo Application Programming Interfaces (APIs), Weibo messages related to COVID-19 were collected with “pneumonia” and “coronavirus” as the keyword with timestamps between 00:00 on January 9, 2020 and 24:00 on February 10, 2020. The following information was extracted: user ID, timestamp (i.e., the time at which the message was posted), text (i.e., the text message posted by a user), and location information.

The original Weibo texts contain interfering information such as http hyperlinks, spaces, punctuation marks, hashtags, and @users. Text filtering was thus necessary to eliminate noise and improve the efficiency of word segmentation. These types of interfering information were removed by regular expression operations (“re” module) in Python (Python Software Foundation, Beaverton, OR, USA). Very short Weibo texts (less than four words) and duplicated Weibo texts were deleted. That left 1,413,297 Weibo messages, including 105,330 texts with geographical location information.

### 2.2. Method

#### 2.2.1. Time Series Analysis

A time series analysis of Weibo texts was used to investigate the temporal diversification of the number of Weibo texts during COVID-19. The original time series of social media data fluctuated in cycles of days [20]. To explore further the temporal trend of Weibo texts, the original time series was decomposed using the Seasonal-Trend decomposition procedure based on Loess (STL), using statistical product and service solutions (SPSS Inc., Chicago, IL, USA) software. As expressed in Equation (1), the time series can be considered the sum of three components: a trend component, a seasonal component, and a remainder in STL:

(1) $$x_{t}=T_{t}+S_{t}+R_{t}.$$

where $x_{t}$ is the original time series of interest. $T_{t}$ is the trend component. $S_{t}$ is the seasonal component. $R_{t}$ is the residual component.

#### 2.2.2. Topic Extraction and Classification

**Figure 1:**
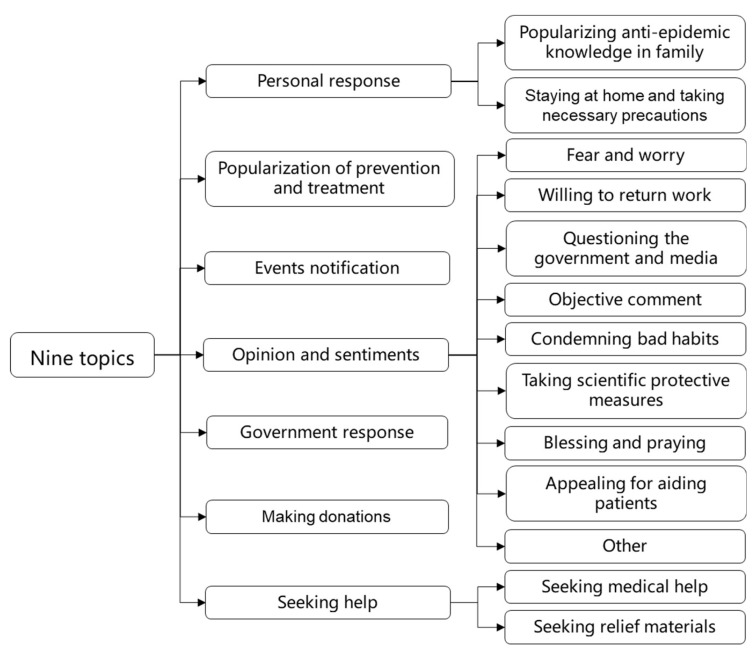
The classification of Corona Virus Disease 2019 (COVID-19) related topics and sub-topics.

A topic extraction and classification model combining the LDA model and the random forest (RF) algorithm was used to hierarchically process COVID-19-related Weibo texts. Existing research has already proved that the LDA model has obvious superiority in identifying semantic topic information from massive text automatically [16,18]. Due to their high computational efficiency in both training and evaluation, in addition to their ability to achieve state-of-the-art results, random forests (RF) are frequently used in text classification [21]. The first step was to mine and generalize the topics from the COVID-19-related Weibo sample using the LDA model. Then, topic extraction results were utilized as training samples for the RF algorithm to classify the Weibo data. As shown in Figure 1, the COVID-19-related Weibo texts were generalized into seven topics: “events notification”, “popularization of prevention and treatment”, “government response”, “personal response”, “opinion and sentiments”, “seeking help”, and “making donations”. A secondary classification was implemented to divide “personal response”, “opinion and sentiments”, and “seeking help” into 13 more detailed sub-topics, including “fear and worry”, “questioning the government and media”, “condemning bad habits”, “objective comment”, “taking scientific protective measures”, “blessing and praying”, “appealing for aiding patients”, “willing to return work”, “staying at home and taking necessary precautions”, “popularizing anti-epidemic knowledge in family”, “seeking medical help”, “seeking relief materials”, and “other”.

**Figure 2:**
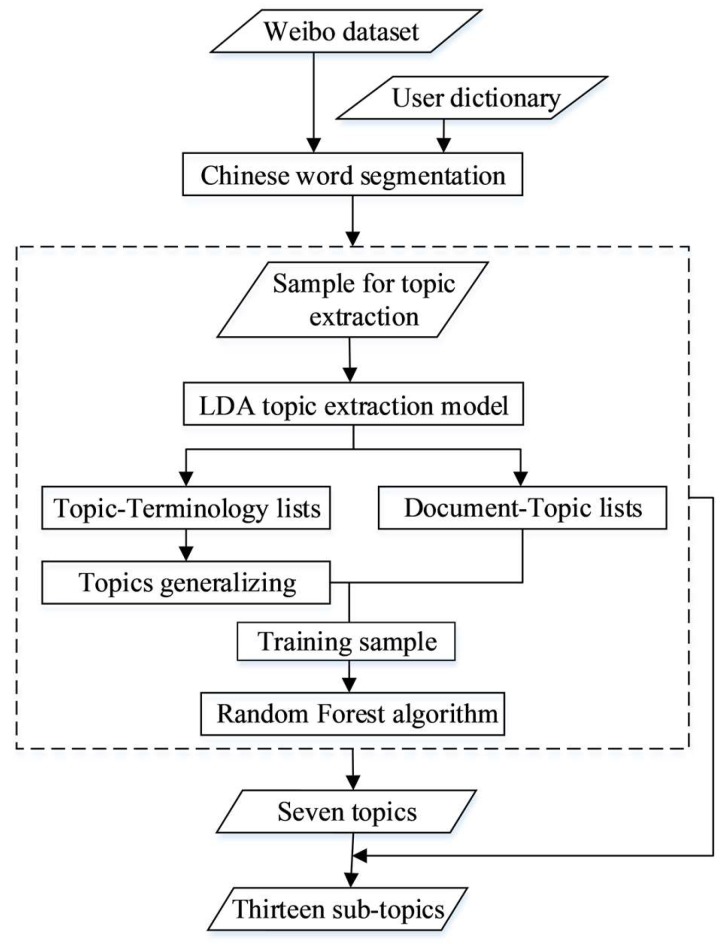
The processes of topic extraction and classification; LDA—Latent Dirichlet Allocation.

The processes of topic extraction and classification are shown in Figure 2, including the steps of word segmentation and topic extraction using the LDA and RF models.

##### Chinese Word Segmentation

Chinese word segmentation was necessary because there are no obvious separators between Chinese words. A Python package for Chinese text segmentation called “Jieba” was utilized. By building a user dictionary including keywords related to COVID-19, the package segmented words efficiently. After this process, the most common stop words that lacked valuable information were removed.

##### LDA Model

LDA is a Bayesian probability model that has three layers—“document-topic-word” [22], with which to identify semantic topic information in large-scale document sets or corpora. In LDA, documents are represented as random mixtures of latent topics, each of which is characterized by a distribution of words [23]. This unsupervised machine learning technique has recently emerged as a preferred method for working with large collections of text documents.

The “Gensim” package in Python was used to implement the LDA model. Through repeated experiments, the optimal number of initial topics was set as 20. The topic-terminology lists obtained from the LDA model contain the vocabularies of each initial topic and the frequency with which those vocabularies occur. The document-topic lists show the probability that each Weibo text is associated with each of the initial 20 topics. We assigned each Weibo text to the topic that it most closely resembled according to the probabilities in the document-topic lists. Based on the topic-terminology lists, 20 topics were generalized into seven (“thirteen” in the secondary classification) by merging similar topics and discarding irrelevant topics.

##### RF Algorithm

The RF classifier is considered a top-notch supervised algorithm in a wide variety of automatic classification tasks [24]. Random forests are a combination of tree predictors, wherein each tree depends on the values of a random vector sampled independently and all trees in the forest have the same distribution [25].

The RF algorithm was used to classify the Weibo texts into different topics. This was implemented using a machine learning package called “scikit-learn” in Python. Based on the document-topic lists, 7000 annotated Weibo texts were used as training samples and 1400 annotated Weibo texts were used as test sets. The number of classification trees (n estimators) was an important parameter for classification accuracy [23]. We used the out-of-bag (OOB) outputs to determine the optimized values of the parameters at 200.

#### 2.2.3. Kernel Density Estimation

Kernel density estimation is generally used to detect the intensity of events by generating a smooth surface using a quadratic kernel function [26]. Let ($s_{1}$,…, $s_{i}$,…,$s_{n}$) be a series of event samples distributed with an unknown density $\hat{\lambda }\left(s\right)$, which can be estimated by Equation (2):

(2) $$\hat{\lambda }\left(s\right)=\overset{n}{\underset{i=1}{\sum }}\frac{1}{\tau^{2}}k\left(\frac{s-s_{i}}{\tau }\right)$$

where k is the kernel function, τ is a smoothing parameter called the bandwidth, that is, the search radius within which to calculate density, and $s-s_{i}$ is the distance between s and $s_{i}$.

To identify the hot spots of Weibo texts, kernel density estimation was performed using ArcGIS software. There are two parameters: the kernel search radius (bandwidth) for calculating the density and cell size for the output raster data. A kernel search radius (100–500 km) was used to analyze spatial characteristics at different scales. A cell size of 5 km was used to show the output raster map.

#### 2.2.4. Spearman Correlation

Spearman’s rank correlation coefficient or Spearman correlation is a nonparametric measure of rank correlation (statistical dependence between the rankings of two variables). It assesses how well the relationship between two variables can be described using a monotonic function. The Spearman correlation coefficient is defined as the Pearson correlation coefficient between the rank variables [27].

For a sample of size *n*, the *n* raw scores $X_{i}$,$Y_{i}$ are converted to ranks $rgX_{i}$, $rgY_{i}$, and Spearman correlation ($r_{s}$) is computed as Equation (3):

(3) $$r_{s}=\rho_{rgX,rgY}=\frac{cov\left(rgX,\;rgY\right)}{\sigma_{rgX}\sigma_{rgY}}$$

where ρ denotes the usual Pearson correlation coefficient, but applied to the rank variables. cov(rgX, rgY) is the covariance of the rank variables. $\sigma_{rgX}$ and $\sigma_{rgY}$ are the standard deviations of the rank variables.

#### 2.2.5. Evaluation of Results

Precision, recall, and the F1-measure were used to evaluate the accuracy of the classification. Precision is the fraction of correctly classified positive items among the total. Recall measures the proportion of actual positives that are correctly identified. The F1-measure is a weighted harmonic mean of precision and recall. Higher values of the F1-measure indicate that the classification method is more effective [28]. Precision (P), recall (R), and F1-measure (F1) are defined as Equations (4)–(6):

(4) $$P=\frac{T_{P}}{T_{P}+F_{P}}$$

(5) $$R=\frac{T_{P}}{T_{P}+F_{n}}$$

(6) $$F1=\frac{P\times R\times 2}{P+R}$$

where $T_{P}$ is the number of correctly classified positive items. $F_{P}$ is the number of incorrectly classified positive items. $F_{n}$ is the number of incorrectly classified negatives.

## 3. Results

### 3.1. Spatial-Temporal Analysis

#### 3.1.1. Time Series Analysis

**Figure 3:**
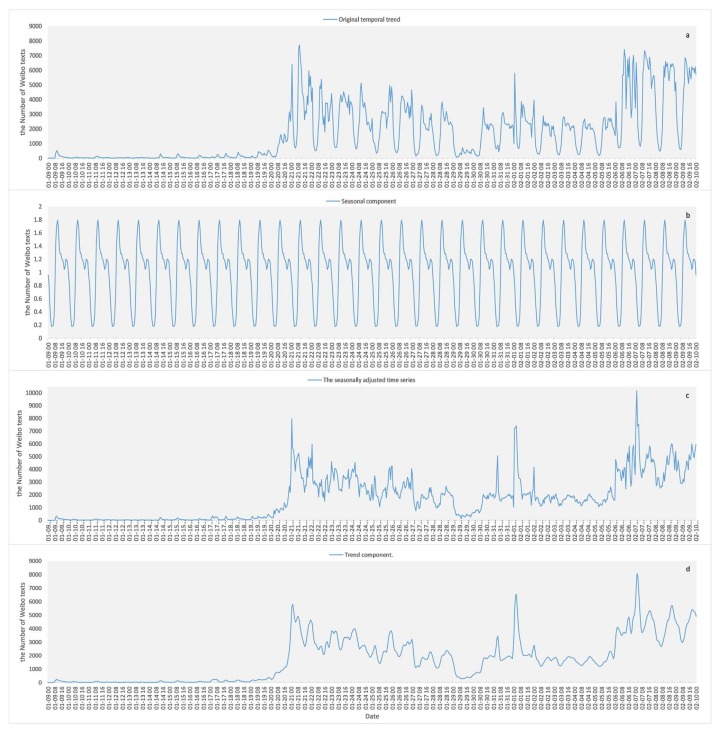
The seasonal trend decomposition of the temporal trends of COVID-19-related Weibo. (**a**) Original temporal series; (**b**) seasonal component; (**c**) seasonally adjusted time series; (**d**) trend component.

The results of the time series analysis of COVID-19-related Weibo texts are shown in Figure 3. Figure 3a shows the original time series of the number of Weibo texts. Split by day, it shows that the lowest point of the Weibo number on the curve for each day appeared at 06:00, after which the curve began to rise sharply. Figure 3b shows part of the cyclical change in the number of COVID-19-related Weibo posts. The lowest point of cyclical change occurred at 06:00 every day, with two daily peaks around 11:00 and 23:00. Figure 3c shows the seasonally adjusted time series, which shows the trend of the number of COVID-19-related Weibo texts after eliminating the seasonal factor. Figure 3d shows the trend component reflecting the trends of the number of COVID-19 related Weibo. After COVID-19 occurred, a slight increase appeared for a short time, then the amount increased sharply on 20 January. The fluctuation reached a peak on 21 January, and then began to decrease but fluctuated until 29 January. The curve rose obviously on 31 January and reached a peak on 1 February. It then steadily fluctuated from 2 February to 5, started to climb on 6 February, and then steadily declined after reaching the highest peak on 7 February.

**Figure 4:**
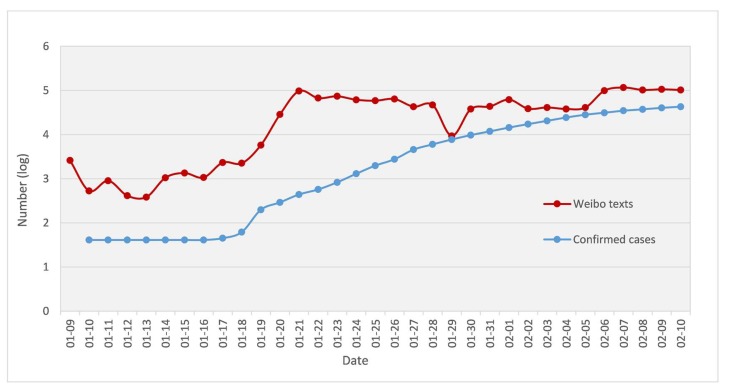
Daily number of Weibo texts and confirmed cases on a Log scale.

As can be seen from Figure 4, the rising trend of daily Weibo numbers and that of the confirmed cases of epidemics is very similar in this early stage. They both rapidly rose around January 19. However, the overall Weibo response was quick and higher than the number of confirmed cases. Since then, the number of confirmed cases continued to rise, but due to the Chinese New Year holiday, Weibo data remained stable (or even slightly decreased) and continued to rise steadily after 29 January in line with the trend of confirmed cases.

#### 3.1.2. Spatial Analysis

**Figure 5:**
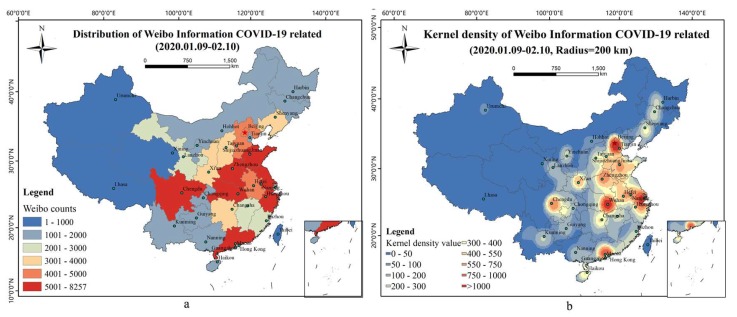
The spatial distribution of Weibo related to COVID-19. (**a**) Distribution of Weibo Information COVID-19 related (2020.01.09–02.10). (**b**) Kernel density of Weibo Information COVID-19 related (2020.01.09–02.10).

The spatial distribution of Weibo related to COVID-19 is shown in Figure 5. The Weibo numbers were mainly concentrated in the east-central parts of China, as shown in Figure 5a. There were more than 5000 Weibo texts in Shandong Province (the capital of Jinan), Hubei Province (capital of Wuhan), Henan (capital of Zhengzhou), Guangdong (capital of Guangzhou), Sichuan (the capital of Chengdu), and Jiangsu (capital of Nanjing), Anhui Province (capital of Hefei), Hebei Province (capital of Shijiazhuang), Beijing, Shaanxi (capital of Xi’an), Liaoning (capital of Shenyang), Hunan (capital of Changsha), and Shanxi (capital of Taiyuan). Figure 5b shows the spatial distribution of the kernel density with a search radius of 200 km, indicating that the high-density areas of Weibo related to COVID-19 were in Wuhan, Beijing, Shanghai, Guangzhou, Chengdu, Xi’an, and Zhengzhou, and presents a continuous trend among the hot points of Wuhan, Beijing, and Shanghai.

**Figure 6:**
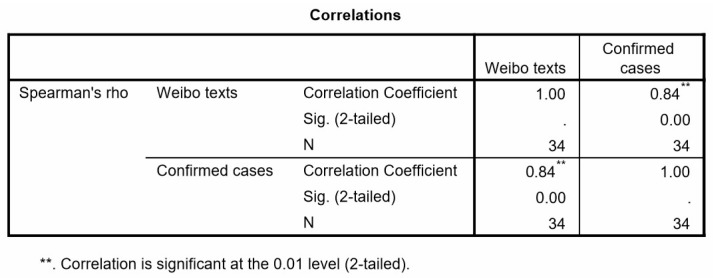
Spearman correlation of COVID-19-related Weibo texts and confirmed cases in provinces.

In order to explore the correlation between public opinion and the epidemic situation, this study used Statistical Product and Service Solutions (SPSS Inc., Chicago, IL, USA) software to perform Spearman correlation analysis on the number of relevant Weibo texts and confirmed cases in provincial level (number is 34). The descriptive statistics of two variables (the number of Weibo texts and confirmed cases in provinces) is shown in Table 1. Figure 6 shows that the Spearman correlation coefficient is 0.84 and significant statistical significance (*p* = 0.00 < 0.01), so public opinion and epidemic situation have a significant positive correlation with a confidence degree of 0.01.

**Figure 7:**
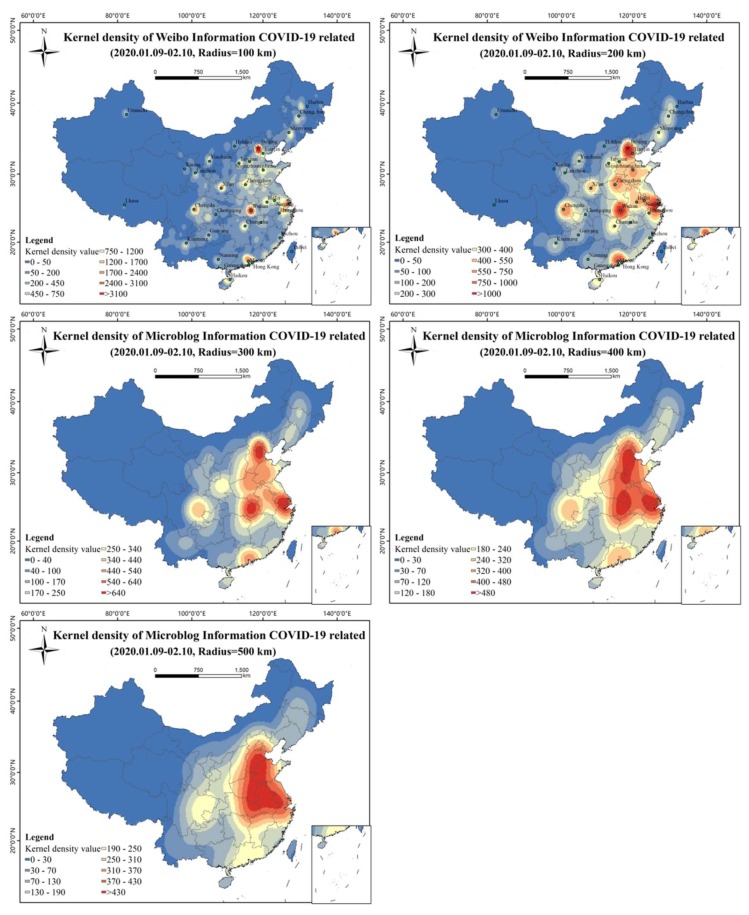
Kernel density maps of COVID-19-related Weibo texts at different scales.

The spatial kernel density characteristics of Weibo texts at different scales can be shown by setting different search radii (Figure 7). The result with a search radius of 100 km shows that Wuhan was the focus center, surrounded by Beijing, Shanghai, Guangzhou, Chengdu, and Xi’an, which were star-shaped and supplemented by prominent Weibo high-value areas of provincial capitals. The result with a search radius of 200 km reflects that Wuhan, Beijing, Shanghai, and Guangzhou are the core, Chengdu, Xi‘an, Zhengzhou, Jinan, and Shijiazhuang are prominent, and the triangular region of Wuhan, Beijing, and Shanghai is in a continuous trend. The result with a search radius of 300 km shows a contiguous regional pattern with core nodes of the Beijing-Tianjin-Hebei junction region, the whole area of the east Hubei province and adjacent province region, and the cross-border region of Jiangsu, Zhejiang, and Anhui as well as two independent core regions of Guangzhou and Chengdu. The result with a search radius of 400 km highlights the contiguous areas in which the cross-border area of Hebei-Shandong, Hubei-Hebei, and the Jiangsu-Zhejiang-Shanghai-Anhui border area are core nodes, and Guangzhou and Chengdu are two independent core areas. The result with a search radius of 500 km shows the core area as a triangular region, with Beijing, Hebei, Shandong, Henan, Hubei, Anhui, Jiangsu, Zhejiang, and Anhui connected, and gradually connected with Guangdong and Sichuan.

### 3.2. Topic Analysis

#### 3.2.1. Topic Description

**Figure 8:**
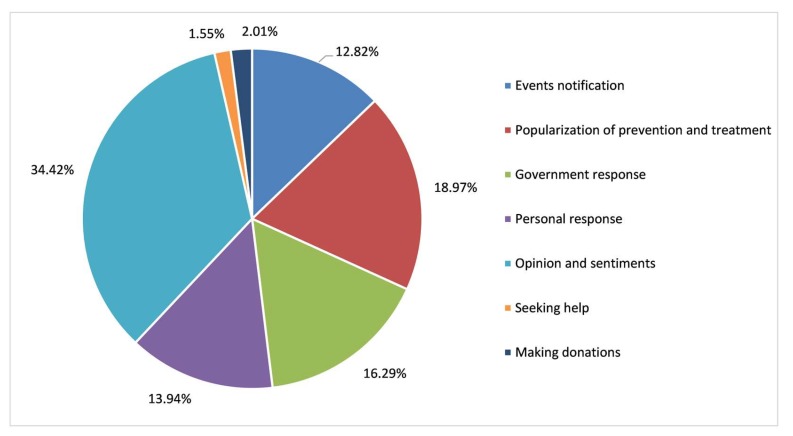
Classification of topics in Weibo texts related to COVID-19.

Figure 8 illustrates the statistical results of the percentage of first-level topics of COVID-19. “Opinion and sentiments” accounted for 34.42% of all topics. “Popularization of prevention and treatment” and “government response” were the second and third most frequent, at 18.97% and 16.29%, respectively. The proportion of “events notification” and “personal response” comprised 13.94% and 12.82%, respectively. “Seeking help” and “making donations” then accounted for 2.01% and 1.55%, respectively.

**Figure 9:**
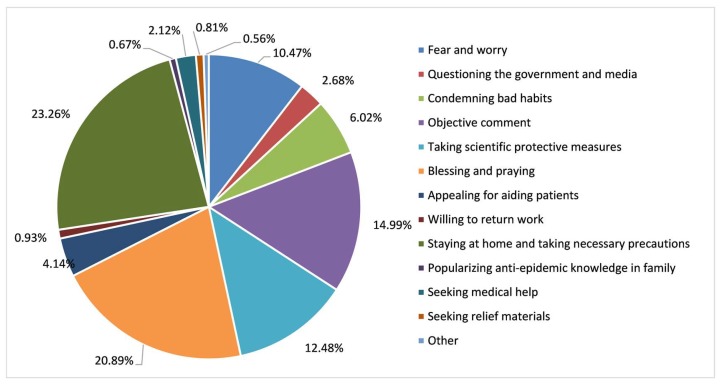
Classification of sub-topics in Weibo texts related to COVID-19.

A more in-depth analysis of the proportions of sub-topics is presented in Figure 9. “Staying at home and taking necessary precautions”, “blessing and praying”, and “objective comment” were the three most widespread sub-topics, accounting for 23.26%, 20.89%, and 14.99% of texts. The proportion of “taking scientific protective measures” and “fear and worry” comprised 12.48% and 10.47%, respectively. This was followed by “condemning bad habits” and “seeking medical help”, which accounted for 6.02% and 4.14%. The proportion of other sub-topics was less than 3%.

After computing precision, recall, and F1-measure values, the classification accuracy of the topics and sentiments is presented in Table 2. For the seven topics, the precision was found to be 83% and F1 was 82%. For the 13 sub-topics, the precision and F1 values were 78% and 76%, respectively.

#### 3.2.2. Temporal Trend of Topics

**Figure 10:**
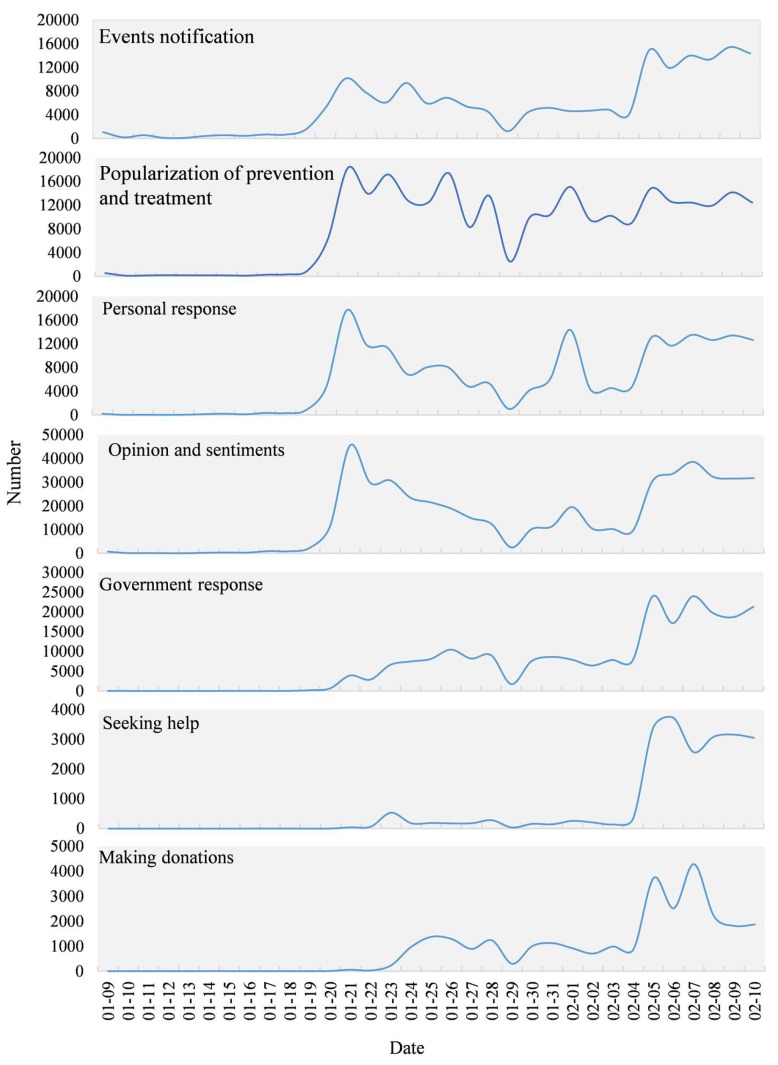
The temporal series of topics during COVID-19.

To display accurate temporal changes in the different topics, the number of Weibo texts for each topic was counted using one-hour time intervals as shown in Figure 10. The topics of “events notification”, “popularization of prevention and treatment”, “personal response”, and “opinion and sentiments” all climbed from 19 January reaching a peak on the 21st. The curve then steadily declined towards the 29th and rose slowly to 1 February. There was a small peak on February 1, then it stabilized and reached a peak again on 5 February. The topics of “government response” and “making donations” started to rise steadily from 20 January, then declined after showing a small peak around 26 January, after which it started to climb on 4 February and reached a peak on 5 February. “Seeking help” started to rise suddenly on 22 January showing a small peak before and after Wuhan was placed under lockdown on the 23rd, then climbing on 4 February reaching a peak on 6 February and then levelling off.

**Figure 11:**
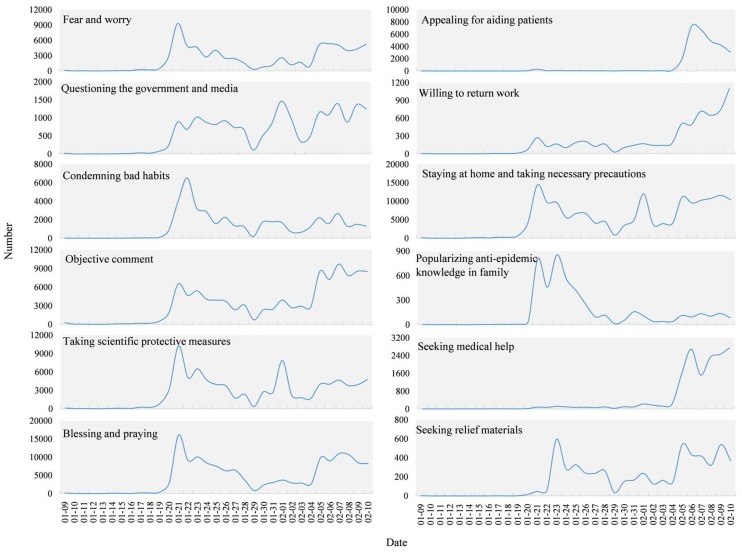
The temporal series of sub-topics during COVID-19.

Figure 11 presents the time series of all sub-topics except “other”. From the perspective of the general trends, the three sub-topics, “questioning the government and media”, “staying at home and taking necessary precautions”, and “taking scientific protective measures” showed a similar variation tendency over time. The numbers of texts on those three sub-topics improved quickly on 20 January and peaked on the 21st, then gradually decreased but fluctuated towards 29 January, rose obviously on 31 January, and reached a peak on 1 February. Since then, the curve has been steadily fluctuating, beginning to rise on 5 February. “Fear and worry”, “objective comment”, and “blessing and praying” climbed from 20 January, reached their peak on 21, fell steadily, then rose again on 5 February and stabilized. “Appealing for aiding patients” and “seeking medical help” suddenly increased from 6 February and reached a peak around 8 February. After that, the “appealing for aiding patients” showed a downward trend, and the “seeking medical help” remained a high concern. “Popularizing anti-epidemic knowledge in family” and “condemning bad habits” both started to climb on 20 January. After reaching a summit on the 21st, the decline since stabilized. “Seeking relief materials” began to rise on 22 January, fell to a peak on 23, then stabilized after rising on 5 February. “Willing to return work” had a slight increase and fluctuation since 20 January and has shown a significant upward trend since 4 February.

#### 3.2.3. Spatial Distribution of Topics

**Figure 12:**
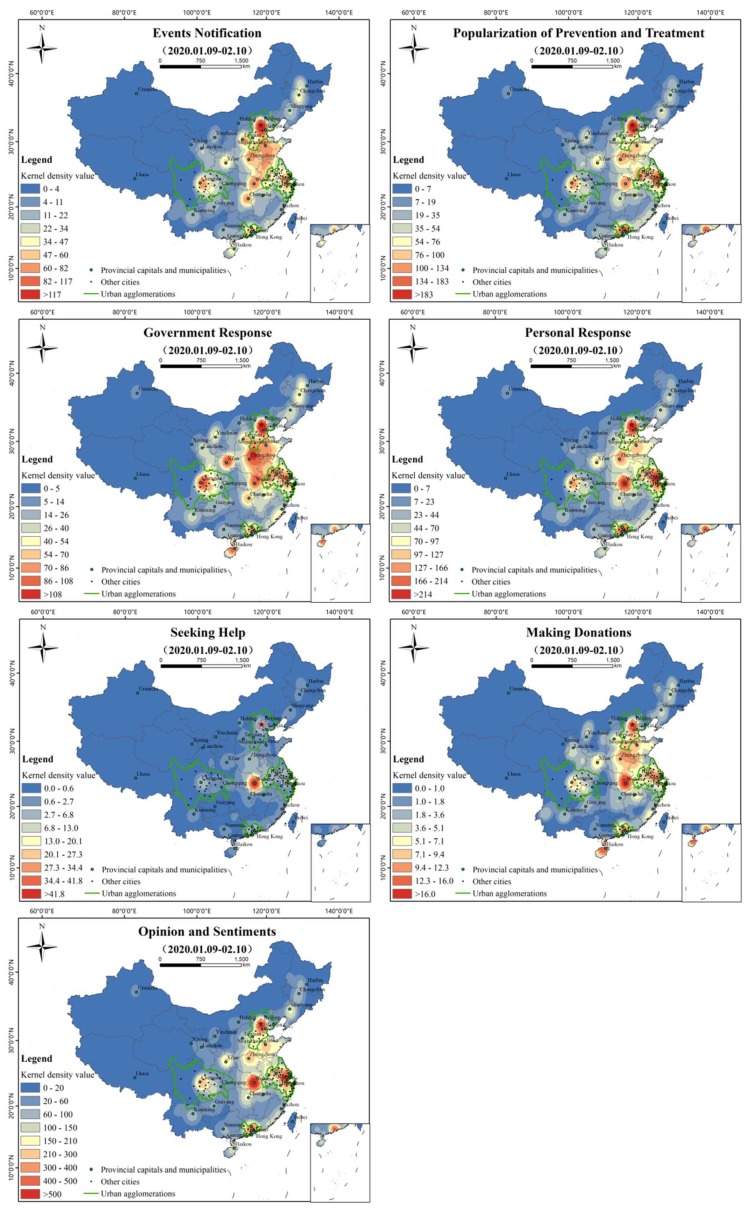
Kernel density analysis of topics (search radius = 200 km).

Kernel density analysis (radius of 200 km) was carried out on Weibo with geographical locations in each topic, as shown in Figure 12. The spatial distribution of “events notification”, “popularization of prevention and treatment”, “government response”, “personal response”, and “opinion and sentiments” is similar to the general characteristics of Figure 5b, forming hot spots in Beijing-Tianjin-Hebei, Shandong, Henan, Hubei, Yangtze River Delta, Sichuan, and Guangdong, but there are differences within the topics. “Events notification” takes Beijing, Wuhan, Shanghai, and Sichuan as prominent high-value areas, and the areas of the Beijing-Tianjin-Hebei cross border area, east Hubei, and the Jiangsu-Zhejiang-Shanghai cross border areas are the main nodes in a continuous pattern. “Popularization of prevention and treatment” is presented with Beijing, Guangzhou, and Shanghai as the prominent high values, supplemented by Wuhan, Chengdu, Hefei, Zhengzhou, and other high-value areas. “Government response” has Beijing, Sichuan, and Xi’an as high values, though Zhengzhou, Wuhan, Changsha, Shanghai, Guangzhou, Haikou, and other cities have responded significantly. “Personal response” is prominently reflected by Beijing, Shanghai, Guangzhou, and Wuhan, with Beijing, Wuhan, and Shanghai as the center and Guangzhou and Chengdu as relatively independent high-value areas. “Opinion and sentiments” was more prominent in high-value areas around Wuhan, followed by the Yangtze River Delta, Beijing-Tianjin-Hebei, and the Pearl River Delta urban agglomeration. “Seeking help” and “making donations” show totally different characteristics. “Seeking help” appears significantly around Wuhan and shows a trend of diffusion to the surrounding areas, especially to the north. “Making donations” has Beijing and Hainan as high values and spreads across the country, but is relatively concentrated in urban areas around Wuhan, the Yangtze River Delta region, Chengdu-Chongqing region, Guangzhou, Zhengzhou, and even Haikou in the south.

**Figure 13:**
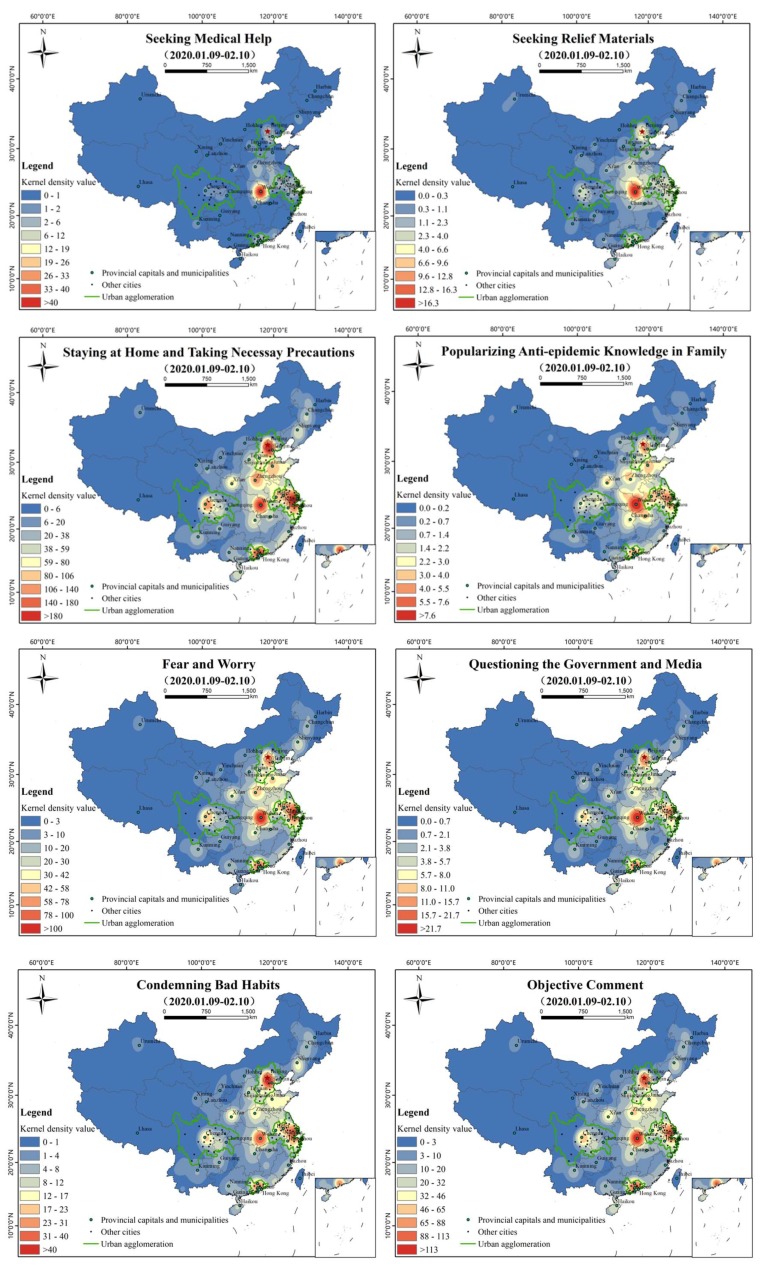

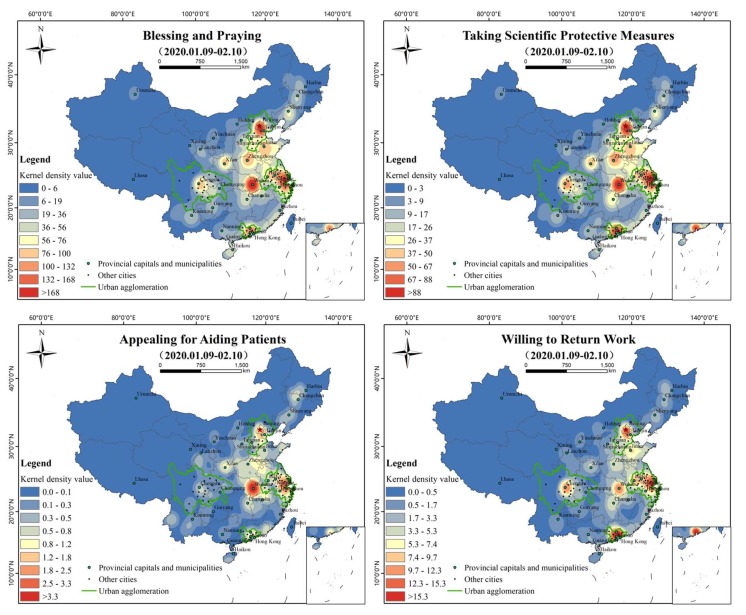
Kernel density analysis of sub-topics (search radius = 200 km).

The spatial distributions of the kernel density estimation of the 13 sub-topics are shown in Figure 13. Except for “appealing for aiding patients”, “seeking medical help”, and “seeking relief materials”, the spatial distribution of most topics is similar to the general characteristics of Figure 5b. “Fear and worry” formed high-value areas in Wuhan, Shanghai, Suzhou, Jiaxing, and other cities. “Questioning the government and media” is mainly reflected in Wuhan, supplemented by the Beijing-Tianjin-Hebei transboundary area, east Hubei, the Jiangsu-Zhejiang-Shanghai neighborhood area, and Guangzhou and Chengdu, two relatively independent high-value areas. “Condemning bad habits” is distributed in dots as a whole. Beijing is a high-value region with prominent dots, and east Hubei, the Jiangsu-Zhejiang-Shanghai cross border area, Guangzhou, and Wuhan are independent high-value regions. “Objective comment” takes Wuhan as a prominent high-value area, supplemented by Beijing, Shanghai, Guangzhou, and other high-value areas. “Taking scientific protective measures” is a prominent spot-shaped high-value area in Beijing, Wuhan, and Shanghai, and the areas within the Beijing-Tianjin-Hebei neighborhood area, east Hubei, the Jiangsu-Zhejiang-Shanghai transboundary areas are the main nodes in a continuous pattern. “Blessing and praying” is centered on the contiguous areas of Beijing, Wuhan, and Shanghai, while Guangzhou, Chengdu, and Zhengzhou are relatively independent high-value areas. “Appeal for aiding patients” takes Wuhan as the center of east Hubei as the high-value area, and Beijing, Shanghai, and the neighborhood area as relatively high-value areas. “Willing to return work” shows that Beijing, Guangzhou, and Shanghai are prominent high-value areas, supplemented by Wuhan, Chengdu, Hefei, Jinan, and other relatively high-value areas. ‘Staying at home and taking necessary precautions’ is led by Wuhan, with Beijing, Wuhan, Shanghai, and Guangzhou as the highlighted high-value areas, and the Beijing-Tianjin-Hebei cross border area, east Hubei, the Yangtze river delta, and the Pearl River Delta as the main nodes, showing a continuous trend. “Popularizing anti-epidemic knowledge in family” is concentrated in Wuhan and its surrounding cities, supplemented by relatively high-value areas such as Beijing, Shanghai, and Guangzhou. “Seeking medical help” and “seeking relief materials” are prominently concentrated in Hubei. “Seeking medical help” appears in Wuhan and spreads to the surrounding area, especially to the east. The overall distribution of “seeking relief materials” and “seeking medical help” showed a similar distribution trend, with Wuhan and its surrounding areas as high-value areas. “Appealing for aiding patients” is mainly distributed in Wuhan, Beijing, Shanghai, and other regions.

## 4. Discussion

### 4.1. Temporal Trend

In terms of the time series analysis, the number of relevant Weibo texts under the COVID-19 event shows a certain periodicity. There are two peaks at which Weibo are sent every day. The peak in the morning usually appears around 11 o’clock, and the peak in the evening usually appears around 23 o’clock. In addition, the number of relevant Weibo has a good correspondence with the development time node of the coronavirus event (shown in Figure 4). Since the characteristics of person-to-person infections for COVID-19 were clarified on 20 January and the central government of China demanded high attention from the public, the number of Weibo texts began to rise significantly, and the fluctuations dropped after reaching a peak on the 21st. By the early hours of 31 January, when the World Health Organization (WHO) announced the epidemic as a “Public Health Emergency of International Concern”, the number of texts had risen markedly, and fell back after reaching its peak on 1 February. On 3 February Huoshenshan Hospital (Wuhan) was officially put into use in response to the epidemic. On the afternoon of 5 February, the online help channel in People’s Daily was opened, and the number of Weibo texts began to climb. When the “whistle-blower” Dr. Wenliang Li passed away on 7 February the number of Weibo reached the highest level, exceeding 110,000 times a day. It then fluctuated, falling back but still at extremely high values.

As shown in Figure 4, there were an increasing number of confirmed cases around January 19 which aroused substantial public concern, and Weibo posts emerged in large numbers. Then, the number of confirmed cases continued to increase while Weibo texts maintained a stable and slightly downward trend, because of the Chinese New Year holiday. After January 29 Weibo texts increased, with fluctuations. Changing trends in public opinion, as expressed on Weibo, reflect actual changes in confirmed cases. Furthermore, other scholars obtained similar findings in different disaster events. Ye found that the developmental trend of dengue disease outbreak events and the trend of the number of Weibo texts was highly correlated [11]. There is a synchronization between daily discussion frequencies on Weibo and the real-world trend of the COVID-19 outbreak. Furthermore, the number of epidemic-related Weibo texts is also influenced by social events, such as the Spring Festival holiday, the seal-off of Wuhan, and the death of public personalities.

Time series analysis of various topics shows that the “events notification”, “popularization of prevention and treatment”, “personal response”, “opinion and sentiments”, and “government response” are similar to the overall trend of Weibo. Since 20 January, they have shown an upward trend during the shock. “Making donations” and “seeking help” are relatively lagging behind. In the early stage of the COVID-19 outbreak, a sudden increase in the confirmed cases caused a shortage of hospital beds. Due to this, patients with suspected cases of COVID-19 had to be in isolation at home. This demonstrated that the planning and allocation of medical resources was under enormous pressure in the early stage of the epidemic. Then, with the establishment of the Huoshenshan and Leishenshan Hospitals (Wuhan) in early February, this situation was relieved but not resolved thoroughly. On 23 January Wuhan was on lockdown, the “making donations” began to rise steadily and rose significantly after 4 February. “Seeking help” started to significantly climb after 4 February and on 5 February, the curve reached its peak when an additional online help channel was opened. The above characteristics indicate that public expressing opinions in emergencies is not random but has periods of silence and noise that are highly volatile and vulnerable to external influences. These results are consistent with previous studies of public responses in emergency situations. The public sentiment in Twitter was highly correlated with external factors, such as the impact from official mass media, important social events, extreme weather, and public holidays [29,30].

### 4.2. Spatial Distribution

The results of spatial kernel density analysis showed that epidemic-related Weibo texts were mainly concentrated in Hubei Province, Beijing-Tianjin-Hebei, the Yangtze River Delta, the Pearl River Delta, Chengdu-Chongqing, and Shandong and Henan. Wuhan, Hubei Province, as the heart of the outbreak of COVID-19, is undoubtedly a hot spot of public concern. Most of the other regions mentioned above are densely populated areas and areas of economic development. In 2018, the proportion of the resident population in urban agglomerations such as Beijing-Tianjin-Hebei, the Pearl River Delta, the Yangtze River Delta, and Chengdu-Chongqing reached 30.8%, and the GDP of these areas accounted for 53.7% of the country. High levels of economic development and population density do not only mean that these regions have a convenient transportation infrastructure network, as these factors may increase the possibility of the epidemic spreading among people more rapidly, which makes epidemic prevention and control more difficult. Improving emergency management and social governance in urban areas during outbreak responses is an issue that should be emphasized.

With regard to the Spearman correlation coefficient, the epidemic situation has a high correlation with the spatial distribution of public opinion. Thus, the spatial distribution of Weibo texts could be related to the severity of the epidemic, the degree of population aggregation, and the level of economic development. These findings are similar to existing research outcomes. For example, the discussions of dengue fever in cyberspace have a strong degree of spatial correlation with real-world epidemic dengue activity [11]. Areas with better socioeconomic conditions generally exhibit higher disaster-related Twitter usage [29].

On a different spatial scale, the spatial kernel density analysis results (Figure 6) indicate that there should be different emergency response strategies at different administrative levels. When the search radius is less than 200 km, the hotspots of Weibo texts are mainly at the city level. When the search radius is 300 and 400 km, there is a significant provincial transboundary area spatial agglomeration of Weibo texts. Thus, enhancing the emergency response at boundary zones among different provinces is the key governance point at the province level. The result with a search radius of 500 km shows a regional spatial hotspot. At the national level, the government should strengthen the response in key urban agglomerations. Considering the differences in the risk levels of COVID-19 among different spatial scales, establishing a hierarchical emergency response mechanism of “region–province–city” is expected to be of substantial significance.

The spatial distribution of each topic is similar to the overall distribution of Weibo, but the aggregation degree of each topic is different. The “events notification” and “popularization of prevention and treatment”, respectively, show high-value areas with Beijing as the core. As the capital and political center of China, Beijing is also the headquarters of many public media. A report on the epidemic can play a role in stabilizing public sentiment. Therefore, it is crucial for the government to take initiatives to make information transparent and to make an appeal for scientific prevention. The distribution of “government response” and “personal response” is similar, but the intensity and scope of the former is higher than that of the latter. In China, local governments responded very strongly and quickly to COVID-19 by following the instructions of the central government. “Seeking help” is concentrated around Wuhan, and “making donations” is more plentiful than “seeking help” in quantity and is distributed globally, reflecting the emergency relief tradition of China: “when trouble occurs at one spot, help comes from all quarters”. “Seeking medical help”, “seeking relief materials”, “appealing for aiding patients”, and “popularizing anti-epidemic knowledge in family” are mainly concentrated around Wuhan, which is related to the severe epidemic situation and the lack of materials in Wuhan. The spatial distribution of ‘willing to return work’ shows that there is a strong willingness to return to work in first-tier cities such as Beijing, Guangzhou, Shanghai, and Chengdu, which have more job opportunities or labor resources.

### 4.3. Topic Discussion

In the early stage of COVID-19 in China, the most expressed topic was ‘opinion and sentiments’, with a proportion of 34.42%. This shows that social media is an important channel through which the public carried out risk perception and shared opinions and emotions during the outbreak of COVID-19. The proportion of “events notification”, “popularization of prevention and treatment”, and “government response” are more than 60%, suggesting that the public are mainly focused on fighting the epidemic, and the timely authoritative information released by the Chinese government is targeted and effective. Since 23 January over 30 provinces successively launched first-level responses to major public health emergencies within three days. As of 30 January, all provinces, including provinces with a few confirmed cases that were substantially distant from Wuhan, had activated a first-level public health emergency response. In the meantime, China strengthened logistics and online platforms to facilitate the online ordering and shopping of goods via contactless delivery. Many cities also issued notices regarding “closed management” to communities in their region, calling for avoiding unnecessary transportation and for working from home. These findings show that the government’s timely release of targeted and effective authoritative information helped eliminate panic and promote the stability of public sentiment. Meanwhile, less than 10% of “seeking help” and “making donations” indicate that more attention should be paid to information directly related to disaster relief: “seeking help” and “making donations” are extremely important, although in small quantities. Paying more attention to vulnerable minority groups should also be considered a crucial aspect of social governance.

In the sub-topics, “staying at home and taking necessary precautions”, “blessing and praying”, “taking scientific protective measures”, and “popularizing anti-epidemic knowledge in family” accounted for more than 50%, indicating that the public opinion in the early stage of COVID-19 was generally positive. In response to the government’s requirements, most people stayed at home and actively took protective measures. Many people paid tribute to medical staff and prayed for the epidemic to pass. Some objective opinions about COVID-19 were also expressed by Weibo users, for example, “objective comment”, “appealing for aiding patients”, and “willing to return work”. However, 19.17% of Weibo texts concerned “fear and worry”, “questioning the government and media”, and “condemning bad habits”, showing negative emotions during the epidemic. People expressed their fears about COVID-19, condemned the consumption of wild animals, and expressed anger at the spread of rumors. When “whistle-blower’’ doctor Li Wenliang passed away, most people expressed their respect and condolences to him, but some expressed their dissatisfaction, considering that the government’s response was too slow or that media reports were untrue. It can be inferred that the timely release and updating of epidemic-related information is an effective measure to avoid public panic and stop the spread of misinformation.

## 5. Conclusions

This study comprehensively analyzed social media data in the early stage of COVID-19 in China and proposed a topic extraction and classification model. The results of the evaluation show that the approach for topic extraction is accurate and viable for understanding public opinions. We obtained seven topics and 13 sub-topics related to COVID-19 from Weibo texts and analyzed their temporal-spatial distributions. (1) The topics with a proportion of more than 60% were “events notification”, “popularization of prevention and treatment”, and “government response”. In the subtopics, “staying at home and taking necessary precautions”, “blessing and praying”, “taking scientific protective measures”, and “popularizing anti-epidemic knowledge in family” was the most-expressed sub-topic. This finding indicates that timely release of information from the government was helpful in stabilizing public opinion in the early stage of COVID-19. (2) The temporal changes in Weibo texts are synchronous with the development of the COVID-19 outbreak. Public opinions during epidemic outbreaks are volatile and vulnerable to external influences. The spatial distribution of COVID-19- related Weibo texts shows a distribution pattern that Beijing-Tianjin-Hebei, the Yangtze River Delta, the Pearl River Delta, and the Chengdu-Chongqing urban agglomeration were significant high-value areas besides Wuhan. This means that the temporal-spatial distribution of opinions was related to the severity of the epidemic, the degree of population aggregation, and the level of economic development. (3) The spatial distribution of public opinions is regionally different and has a scale feature, exhibiting aggregation characteristics in cities, provincial border areas, and key urban agglomeration regions. Though in small quantities, the “seeking help” and “making donations” topics can directly provide support for emergency response and post-disaster management. It is suggested that the government should strengthen the public opinion response and epidemic prevention and control in the key epidemic areas, urban agglomerations, and transboundary areas at the province level; in addition, it should formulate accurate response countermeasures following the public’s demands in controlling the crisis. This study contributes to existing research on uncovering knowledge regarding emergencies from social media by presenting a reliable approach to mining people’s detailed opinions about COVID-19. The findings of this research could provide a rapid situational assessment, help decision makers to better understand public opinions toward COVID-19, and support analysts in planning and executing appropriate resource allocation.

Nevertheless, this study has some limitations. First, the specific reasons for the temporal-spatial distribution of COVID-19-related Weibo texts need further exploration with more information. Second, the paper only analyzed texts from social media, while other content, such as pictures and videos in blogs, may also be informative. Third, there are manipulated opinions on social media (e.g., “fake news” and “troll opinions”). Thus, the better quantification of the noise caused by manipulated opinions needs further investigation. In addition, reducing the noise caused by manipulated opinions in social media data needs to be explored further. With COVID-19, which has been characterized as a pandemic by WHO [31], we will continually acquire new data from Weibo, train and improve the model, analyze the changes and driving mechanisms of public opinion, and provide active references for governmental responses.

## Figures and Tables

**Table 1:** The descriptive statistics of Weibo texts number and confirmed cases number in provinces.

Variables	Minimum Value	Maximum Value	Mean Value	Standard Deviation
Weibo texts number	26	8257	2902.03	2270.665
Confirmed cases number	1	31,728	1256.91	5395.346

**Table 2:** Evaluation results of topic classification.

	Topics	Sub-Topics
Precision (P)	83%	78%
Recall (R)	82%	77%
F1	82%	76%
